# Vascular endothelial growth factor C treatment for mouse hind limb lymphatic revascularization

**DOI:** 10.1002/vms3.151

**Published:** 2019-02-11

**Authors:** Juliana S. P. Ferrão, Antenor P. Bonfim Neto, Vanessa U. da Fonseca, Liza M.M.de C. Sousa, Paula de C. Papa

**Affiliations:** ^1^ Department of Surgery School of Veterinary Medicine and Animal Science University of São Paulo São Paulo Brazil; ^2^ Health Science Institute Paulista University São Paulo Brazil

**Keywords:** Hind limbs, Lymphatic disorders, Mouse, Revascularization, Vascular Endothelial Growth Factor C

## Abstract

Spontaneous lymphatic revascularization is a challenge and the establishment of new therapeutic strategies may improve life quality for patients suffering from lymphatic disorders. This study was designed to verify if VEGFC treatment improves lymphatic vascularization in a time‐dependent manner in mouse hindlimb (HL) after resection of the inguinal lymph node. Lymphatic vascular density (Vv) and length (Lv) were evaluated by stereology after immunohistochemistry. The control Group (CG) was not manipulated but received saline instead of VEGFC treatment. The surgery Group (SG) had the left inguinal lymph node resected but did not received VEGFC treatment. VEGFC Treated Group (TG) had the node resected and received VEGFC treatment. VEGFC and VEGFR3 local expression were assessed by qPCR. There was an effect of time over Vv and Lv in the SG and significant difference between CG and SG in the regions studied (proximal, medium and distal regions) of the left HL (LHL). The Lv showed significant difference between CG and SG only in the medium region. The Vv and the Lv for TG were higher than the other groups. VEGFC and VEGFR3 gene expression presented time effect in all regions of the LHL for SG and TG. Both VEGFC and VEGFR3 gene expression presented significant difference between CG and SG, between SG and TG and between CG and TG. This study showed significant decrease in lymphatic vascularization in the left hindlimb of mice after surgical removal of the inguinal lymph node and adjacent lymphatic vessels. Exogenous VEGFC could recover lymphatic vascularization through stimulating neolymphangiogenesis.

## Introduction

The lymphatic system is indispensable for the collection and cycling of tissue‐extravasated fluids, macromolecules and immune cells into the bloodstream (Guo *et al*. 2009; Sleeman *et al*. 2009; Tammela & Alitalo 2010; Schulte‐Merker *et al*. 2011; Marchiò *et al*. 2013; Blum *et al*. 2014; Kim & Jin 2014). In recent years, there was a growing interest in applying the principles of molecular therapy for lymphatic disorders (An & Rockson 2004; Nakamura & Rockson 2008; Shin & Rockson 2008). In particular, the identification of the molecular components of lymphatic development has made it possible to generate molecular models for lymphangiogenesis (Szuba & Rockson 1998; Yoon *et al*. 2003; An & Rockson 2004; Cheung *et al*. 2006; Saito *et al*. 2006; Aschen *et al*. 2014; Kim & Jin 2014).

While the contributory role of lymphangiogenesis induced by growth factors remains controversial (Goldman *et al*. 2005), Jin *et al*. (2009) and others (Szuba & Rockson 1998; Karkkainen *et al*. 2001; Yoon *et al*. 2003; Saaristo *et al*. 2004, 2006; Cheung *et al*. 2006; Tammela *et al*. 2007; Aschen *et al*. 2014; Kim & Jin 2014) demonstrated the therapeutic benefit of increased VEGFC in a variety of small animal models of anatomical and functional deficiencies of lymphatic vessels.

Therefore, we aimed to evaluate the effect of surgical removal of the inguinal lymph node and lymphatic vessels in mice left hindlimb, as well as to compare the lymphatic revascularization time between our study groups using exogenous VEGFC.

## Material and methods

### Experimental design

**Figure 1:**
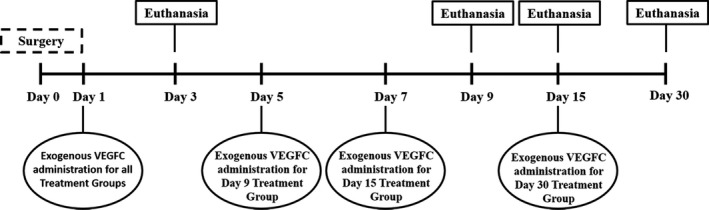
Timeline of the study. Treatment and euthanasia of the Control, Surgery and Treatment Groups. Day 0 is the surgery day. On Day 1, all the Treatment Groups received exogenous VEGFC administration. On Day 5, the Treatment Group euthanized on Day 9 received exogenous VEGFC administration. On Day 7, the Treatment Group euthanized on Day 15 received exogenous VEGFC administration. On Day 15, the Treatment Group euthanized on Day 30 received exogenous VEGFC administration. On Days 3, 9, 15 and 30 animals from Control, Surgery and Treatment Groups were euthanized.

The study was approved by the Ethics Committee for the use of Animals of the School of Veterinary Medicine and Animal Science of the University of São Paulo (number 2289). This study used 52 Balb/C male mice aged 2 months‐old, weighing around 20 grams of weight. Three groups were established: 1 – Control Group (CG; *n* = 12) – animals were not subjected to the inguinal lymph node resection; 2 – Surgery Group (SG; *n* = 20) – animals were submitted to node resection and; 3 – VEGFC Treated Group (TG; *n* = 20) – animals were submitted to node resection and received exogenous VEGFC (Cys156Ser, R&D Systems Inc., Minneapolis, MN, USA) intraperitoneally (0.1 mg mL^−1^) 1, 5, 7 and 15 days after surgery. All mice were euthanized at the same time points (3, 9, 15 and 30 days after housing), but with different numbers of animals in each group, as CG with three mice (total of 12 animal), SG with five mice (total of 20 animals) and VEGFC TG with five mice (total of 20 animals) (Fig. 1).

For surgical procedures, mice were anesthetized intraperitoneally with a mix of ketamine (0.33 mg kg^−1^) and xylazine (0.67 mg kg^−1^) diluted in MiliQ water. A vertical incision was made in the left inguinal region, where the left inguinal lymph node was identified. These were resected without damaging large peripheral blood vessels (Liu *et al*. 2008). During the postoperative period, morphine sulfate (5 mg kg^−1^) was administered subcutaneously every 12 h for 24 h.

The mice were euthanized by an intraperitoneally injection with high concentration of ketamine and xylazine solution. After euthanasia, inguinal skin and all muscular tissue of proximal, medium and distal thirds of the left hindlimb (LHL) were collected for evaluation of Vv and Lv using immunohistochemistry followed by stereological analyses. We also performed quantitative real time PCR (qPCR) for VEGFC and its receptor VEGFR3.

Material was fixed in 4% buffered formalin solution for immunohistochemistry analyses or in liquid nitrogen and then kept at −80°C until further processing.

### Immunohistochemistry for VEGFC and VEGFR3

Samples of inguinal lymph node from both hindlimbs were preserved in buffered formalin for 24 hours, and then embedded in paraffin. Immunohistochemical staining was performed on 15 5 *μ*m sections of each LHL region from all 52 animals studied, as described previously (Ramos‐Vara 2011). Primary antibodies used were anti‐VEGFC (bs‐1586R, Bioss Inc., Woburn, MA, USA) and anti‐VEGFR3 (E‐3, sc‐514825, Santa Cruz Biotechnology, Inc., California, USA). Negative controls were set up using IgG. Rat liver was used as a positive control (Zhuo *et al*. 2012). Observation of immunostaining was made by the same person on the same day. Samples from all groups were run in the same test together with positive and negative controls. The dilution of 1:400 was used for VEGFC antibody and of 1:200 for VEGFR3 antibody.

### Lymphatic vessel stereology

For stereological analysis, samples were collected from the LHL of each animal and processed for immunohistochemical detection of VEGFC to visualize the lymphatic vessels.

Images (2100 images of the LHL for the SG and TG, and 1260 images of the LHL for the CG, totalizing 3360 images) were analysed after superposition of the test area by the Stepanizer software (Tschanz *et al*. 2011). Thus, lymphatic vessels were counted in a known area, called the test area (T_A_). To avoid the overestimation of the vessels, two exclusion (solid) and two inclusion (dotted) lines were adopted. All vessels observed on the exclusion line or touching it were not counted, whereas the vessels observed on the inclusion lines or touching it were counted.

The variable lymphatic vascular volume density (Vv), expressed as a percentage (%) was estimated according to the formula Vv= Pp/P_T_ (Pp ‐ number of test points which touch lymphatic vessels; P_T_ ‐ total number of test points in the system test [100]).

The variable lymphatic vascular length density (Lv), expressed in mm/mm^3^ was estimated according to the formula Lv = 2^(N/A^_T_^)^ (N ‐ number of vessels within the test area; A_T_ ‐ test area [99.404.4586 *μ*m^2^]).

### Quantitative real time PCR (qPCR)

Real‐time PCR (qPCR) was performed as described previously (Tse & Capeau 2003). The primers used were: VEGFC (Mm01202432_m1), Antisense CACCATCAAACATGCAGTTGTTACA; VEGFR3 (Mm00433354_m1), Antisense TCCACCTCCATGTTTGAGGACTATC, and the reference gene beta‐actin (Mm00607939_s1), Antisense ACTGAGCTGCGTTTTACACCCTTTC (Life Technologies, Foster City, California, USA).

### Statistical analysis

Data were analysed for normality of distribution by the Anderson‐Darling normality test (InStat, California, USA). The data were normally distributed, therefore parametric, and hence One Way ANOVA was applied followed by the Tukey‐Kramer posttest for multiple comparisons, (GraphPad Prism 5 Software Inc., San Diego, USA). To compare two means, Student *t*‐test (InStat, California, USA) was performed. The results were presented as mean ± standard deviation and were considered significant when *P* < 0.05.

## Results

### Immunohistochemistry for VEGFC and VEGFR3

**Figure 2:**
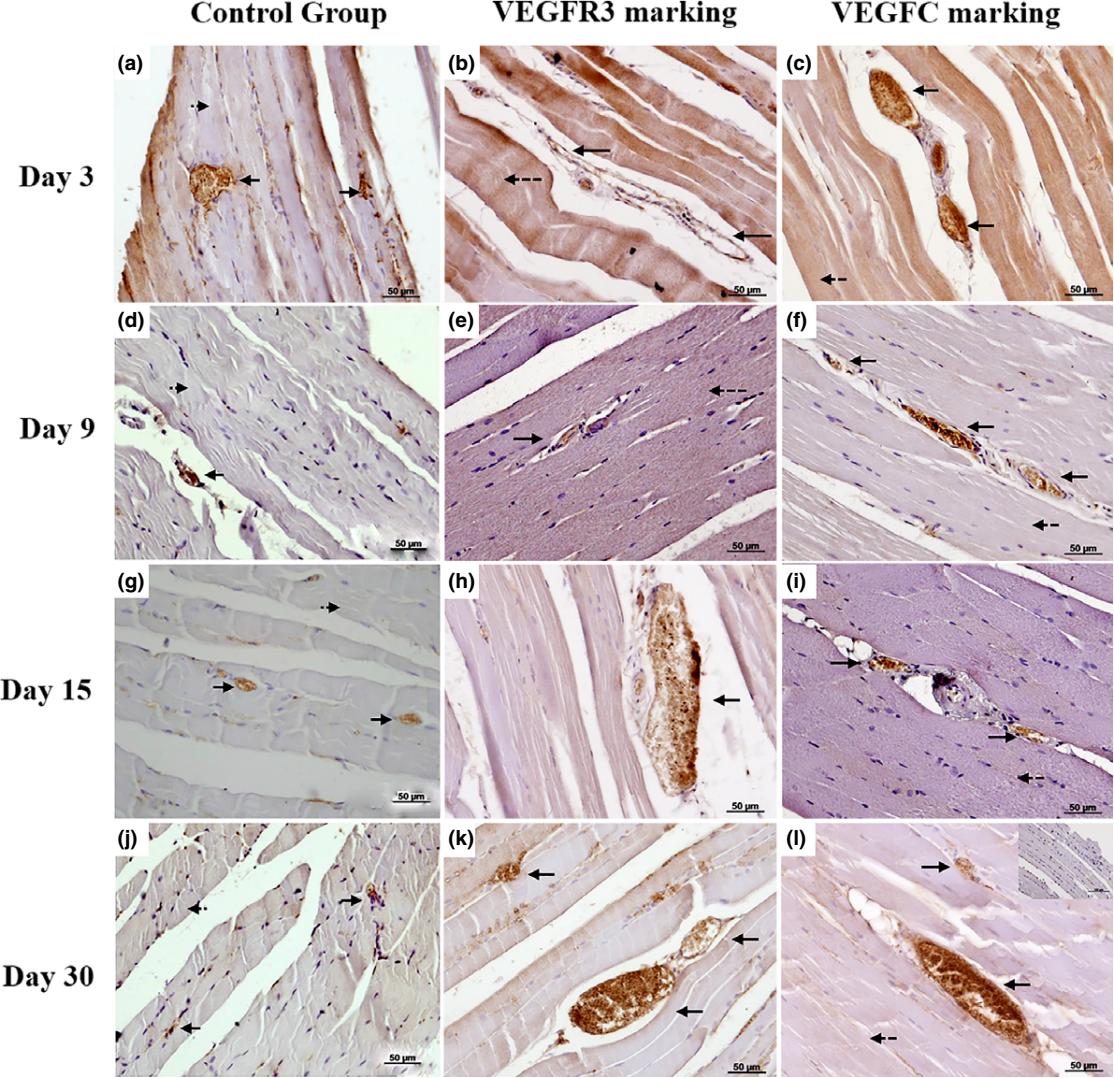
Immunohistochemistry for VEGFC and VEGFR3. Photomicrographs of VEGFC (dilution 1:400) and VEGFR3 (dilution 1:200) immunostaining in the left hind limb muscles of mice representative of either five animals per treatment group (TG) or three animals per control group (CG). Mice were euthanized 3, 9, 15 and 30 days after inguinal lymphnode removal and treatment group received exogenous VEGFC. Counterstaining with haematoxylin. (a, d, g and j) VEGFC staining in CG in mice euthanized on days 3, 9, 15 and 30 after housing, respectively; (b, e, h and k) VEGFR3 staining in the Proximal Region of the left hind limb, 3, 9, 15 and 30 days after surgery, respectively in mice treated; (c, f, i and l) VEGFC staining in the Proximal Region of the left hind limb, 3, 9, 15 and 30 days after surgery, respectively in mice treated with exogenous VEGFC. The arrows indicate the lymphatic vessels, dotted arrows indicate Muscle Tissue. Insert in (l) corresponds to the negative control. Bars of 50 *μ*m.

When comparing immunohistochemical staining for VEGFC and VEGFR3 in the Treatment Group with the other groups (Control and Surgery Groups), we observed qualitatively a more intense positive staining in the different regions of the left hindlimb (Fig. 2).

There was an increased intensity of immunostaining of VEGFC and VEGFR3 over the studied period (3, 9, 15 and 30 days) in the Surgery and Treatment Groups comparing to Control Group (Fig. 2).

### Lymphatic vascular volumetric density (Vv) and lymphatic vascular length density (Lv)

**Figure 3:**
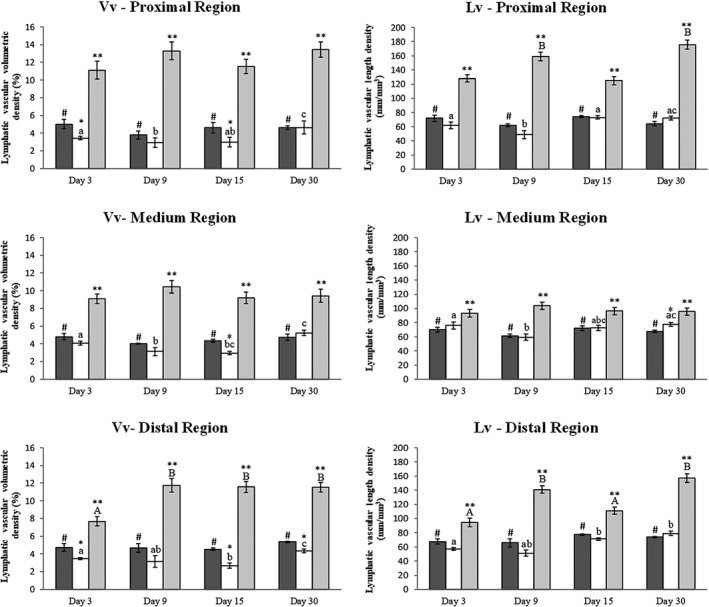
Lymphatic vascular volumetric density (Vv) and Lymphatic Vascular length density (Lv) in the left hind limb of mice. Lymphatic vascular volumetric density (Vv) and Lymphatic Vascular length density (Lv) in the left hind limb of mice. Graphs showing comparison of Vv and Lv between the Control (dark gray columns), Surgery (white columns) and Treatment (light gray columns) groups of animals euthanized on days 3, 9, 15 and 30 after surgery. The bars represent the mean ± standard deviation. Asterisk (*) indicates significant difference (*P* < 0.05) between the Surgery and Control groups in the same observation time, (**) indicate significant differences (*P* < 0.05) between Surgery and Treatment groups in the same observation time, (#) indicates significant difference (*P* < 0.05) between the Treatment and Control groups at the same time of observation, different lowercase letters indicate the effect of time on the Vv and Lv in the Surgery groups and different capital letters indicate the effect of time on the Vv and Lv in the Treatment groups.

The Lymphatic Vascular Volumetric Density (Vv) presented time effect in all the three regions (proximal, medium and distal) of the LHL for the Surgery Group (*P* = 0.0073), once the Vv was lower at Day 09 and higher at Day 30 (Fig. 3).

The Vv presented a significant difference between Control and Surgery Groups in all regions of the LHL (proximal region: *P* < 0.0361; medium region: *P* = 0.0022; and distal region: *P* < 0.0107), with the means of the Surgery Group always lower than the Control Group. The Vv for the Treatment Group was higher than the other groups in all the three regions of the LHL (proximal region: *P* < 0.0001; medium region: *P* < 0.0003; and distal region: *P* < 0.0012).

The Vv for the Treatment Group only changed with time in the distal region of the LHL, with the means on Days 09, 15 and 30 were higher than Day 03 (*P* < 0.05) (Fig. 3).

The Lymphatic Vascular Length Density (Lv) showed a time effect in all the three regions studied for the Surgery Group, with the means being higher on Days 03, 15 and 30 in the proximal region, in Days 03 and 30 in the medium region, and Days 15 and 30 in the distal region (PR: *P* < 0.05; MR: *P* < 0.05; and DR: *P* < 0.01). For the Treatment Group, the Lv showed an effect of time in the proximal and distal regions, with the means higher in both the Day 09 and 30 samples (PR: *P* < 0.001; and DR: *P* < 0.01).

The Lv showed significant difference between Control and Surgery Groups only in the medium region of the LHL on Day 30, with the mean of the Surgery Group being higher than the Control Group (*P* = 0.0302). The Lv for the Treatment Group was higher than the other groups in all the three regions of the LHL (proximal region: *P* < 0.002; medium region: *P* < 0.036; and distal region: *P* < 0.005) (Fig. 3).

### qPCR for VEGFC and VEGFR3 in mouse hind‐limb

**Figure 4:**
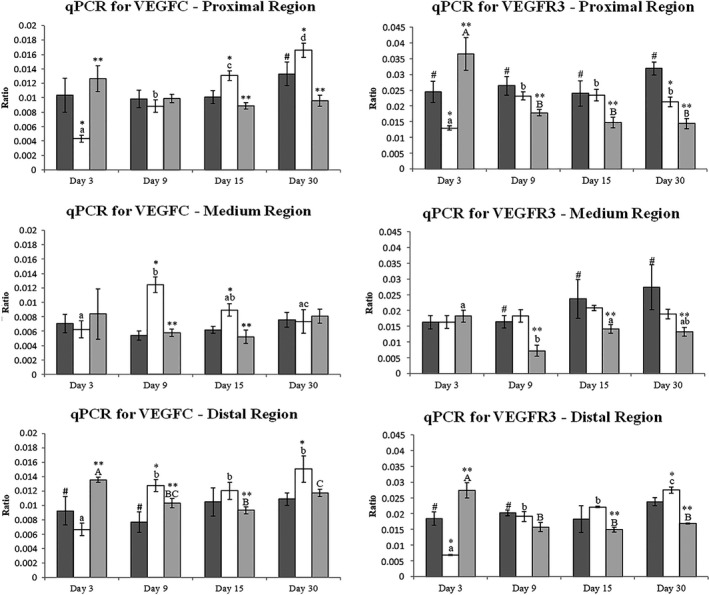
VEGFC and VEGFR3 gene expression of the left hind limb of mice. The graph shows the comparison between the Control (dark gray columns), Surgery (white columns) and Treatment groups (light gray columns) on days 3, 9, 15 and 30 after surgery with and without treatment. The bars represent the mean ± standard deviation. Asterisk (*) indicates significant difference (*P* < 0.05) between the Control and the Surgery groups on the same observation time, (**) indicate significant difference (*P* < 0.05) between Surgery and Treatment groups at the same time observation, (#) indicates significant difference (*P* < 0.05) between the Treatment and Control groups at the same time of observation. Lowercase letters indicate the effect of time on the Surgery group during the period of study and capital letters indicate the effect of time in the Treatment group during the study period.

VEGFC gene expression showed an effect of time in all the three regions of the LHL for the Surgery Group. In the proximal region its expression increased progressively throughout the study (*P* < 0.01). In the medium region, VEGFC expression increased from Day 03 to Day 09 and then decreased progressively until Day 30 (*P* < 0.01). In the distal region its expression increased from Day 03 to Day 09 and from Day 15 to Day 30 (*P* < 0.01). The Treatment Group showed an effect of time only in the distal region of the LHL (*P* < 0.05), decreasing along the period of study from Day 03 to Day 15 and then slightly increasing to Day 30 (Fig. 4).

For VEGFC gene expression Surgery Group demonstrated higher expression on Days 15 and 30 (*P* = 0.0405), and lower expression on Day 03 (*P* = 0.0405; Day 9 *P* > 0.05) when compared with Control Group. In the medium region Surgery Group showed gene expression higher than in the Control Group on Days 09 and 15 (*P* = 0.0087) (Days 3 and 30 *P* > 0.05). In the distal region the Surgery Group showed higher gene expression than the Control Group in Days 09 and 30 (*P* = 0.0415) (Days 03 and 15 *P* > 0.05). There was also significant difference between the Surgery and Treatment Groups in the proximal region in Days 03, 15 and 30 (*P* = 0.0005), in the medium region in Days 09 and 15 (*P* = 0.0160), and in the distal region of the LHL on Days 03, 09 and 15 (*P* = 0.0413). VEGFC gene expressions showed significant difference between Control and Treatment Groups for the proximal region on Day 30 (*P* = 0.0180) and distal region on Day 03 (*P* < 0.0239) (Fig. 4).

The VEGFR3 gene expression showed a time effect in all the regions of the LHL for the Treatment Group. In the proximal region, the mean was higher on Day 03 (*P* < 0.001) and then it suffered a decrease, remaining statistically similar in Days 09, 15 and 30; in the medium region, initially the mean decreased from Day 03 to Day 09, and then increased on Day 15 (*P* < 0.0001); and in the distal region (*P* < 0.001), which behaved as the proximal region. For the Surgery Group, the effect of time was observed in the proximal region (*P* < 0.01), with an increase from Day 03 to Day 09 and no further change through the remaining days of study; and in the distal region (*P* < 0.001), with an increase in the mean from Day 03 to Day 09, remaining statistically similar until Day 15, and increasing once more on Day 30.

The VEGFR3 gene expression was significantly different between Control and Treatment Groups and between Surgery and Treatment Groups. In the proximal region, the Treatment Group presented the mean was only higher at Day 03 (*P* = 0.0232 for the comparison between Control and Treatment Groups; and *P* = 0.0059 for the comparison between Surgery and Treatment Groups); in the medium region, the Treatment Group means are minor than the Control and Surgery Groups (*P* = 0.0406 for the comparison between Control and Treatment Groups; and *P* = 0.0268 for the comparison between Surgery and Treatment Groups); in the distal region, the Treatment Group means behave similarly to the proximal region (*P* = 0.0447 for the comparison between Control and Treatment Groups; and *P* < 0.0001 for the comparison between Surgery and Treatment Groups). The VEGFR3 gene expression presented significant difference between Control and Surgery Groups in the proximal region in Days 03 and 30, with the means of the Control Group higher than the Surgery Group (*P* < 0.0006); and in the distal region in Day 03, with the Control Group mean higher (*P* < 0.0001) and Day 30, and with the Surgery Group mean higher (*P* = 0.0289) (Fig. 4).

## Discussion

Our findings show it is possible to use protein therapy with exogenous VEGFC in areas with lymphatic insufficiency. One treatment with exogenous VEGFC proved to be effective for lymphatic revascularization over a short period of time after lymph node resection. Therefore, it can be used to study and better understand of lymphoedema and lymphatic revascularization, to allow innovative future techniques that may bring comfort and welfare to patients, animals and humans who suffer from lymphatic problems. These observations may have direct implications for future therapeutic approaches for both lymphatic vascular diseases and to cancer metastases (Sleeman *et al*. 2009).

In this study, the process of neoformation of lymphatic vessels was confirmed by observing the revascularization in the different regions of the mouse hindlimb, by assessing both Lymphatic Vascular Volumetric Density (Vv) and Lymphatic Vascular Length Density (Lv). As stated by Mandarim‐de‐Lacerda (2003), who demonstrated that stereological studies show advantages over qualitative studies, as a quantitative tool has numerical results and is not subjective. In our study, it was clear that the removal of the inguinal lymph node affected lymphatic vascular volume and length of the mouse hind‐limb, and that the treatment with exogenous VEGFC was important in the neolymphangiogenesis. Liu *et al*. (2008) showed that VEGFC reduced lymphoedema efficiently via lymphangiogenesis and lymphatic enlargement, suggesting a role of VEGFC in the growth and maintenance of lymphatic vessels. According to Kuwahara *et al*. (2013), intramuscular injection of exogenous VEGFC can enhance the recovery of blood flow in ischaemic limbs and the function of walking, indicating that enhancing lymphatic drainage and venous return accelerates the recovery of hind‐limb ischaemia. The restoration of lymphatic flow is benefited by good tissue healing, manual lymphatic drainage and muscle contraction (Jin *et al*. 2009) and potentially the use of VEGFC, as evidenced by this study.

The proximal region of the LHL showed values of Vv and Lv higher than the other regions of the left hind‐limb for the Treatment Group, indicating that in this region lymphangiogenesis may have been more effective, or even that in this region the need for the presence of lymphatic vessels is higher than in other regions (Hellingman *et al*. 2010; Kochi *et al*. 2013). This difference can also be attributed to the fact that the proximal region of the normal left hindlimb has a greater blood and lymphatic supply, since all the lymph present in the limb must be transported to and through this region. Guo *et al*. (2009) used TNF‐Tg mice as a model of chronic inflammatory arthritis and demonstrated that blockade of VEGFC/VEGFR3 signalling by VEGFR3 neutralizing antibody reduces lymphangiogenesis and lymphatic drainage. Another possibility may be the proximity of this region to the application site of the exogenous VEGFC, since the application was performed intraperitoneally near the region of the surgical wound.

Similar to this study, Jin *et al*. (2009) demonstrated that the ameliorative effect of VEGFC augmentation is evident in the murine model through the positive microvascular remodelling that characterizes the lymphoedema in that model, and by its clear resolution following exogenous VEGFC administration. Using a porcine model, Saaristo *et al*. (2012) found that lymphatic regeneration after microsurgical lymph node transfer can be significantly augmented by exogenously delivered VEGFC. This study also demonstrated significant differences in the variables Vv and Lv in the regions of the left hindlimb between the Control and Surgery Groups; the Surgery Group always exhibited Vv and Lv smaller than their respective Control Group. However, it was noted that an increase in Vv and Lv values throughout the study period could be indicative of spontaneous lymphatic revascularization (lymphangiogenesis), since healthy animals show increased capacity of tissue regeneration. Using lymphoscintigraphy, Aschen *et al*. (2014) found progressive and statistically significant increases in technetium‐ 99m uptake in transplanted nodes, with peak values approximating nonoperated controls, demonstrating an increase in lymphatic function. The Control Group did not differ depending on the day of euthanasia, which was to be expected, since these animals were not manipulated.

Treatment with VEGFC showed a decreased VEGFC and VEGFR3 gene expression in the sites affected by the surgery and an increase in the number of lymphatic vessels. The VEGFC and VEGFR3 gene expression analysis revealed that in the different regions of the LHL, the Surgery Group showed increased gene expression throughout the study period, whereas gene expression for the Treatment Group showed a small decrease. This could suggest that the animals submitted to surgery began to express those proteins gradually, in order to increase the lymphatic vascularization (revascularization) to compensate for the loss of drainage due to surgical removal of the inguinal lymph node and adjacent lymphatic vessels. According to Kazenwadel *et al*. (2012), endogenous VEGFC has effects on important cellular functions for lymphangiogenesis, promoting lymphatic vascular tube formation. However, although exogenous VEGFC gene expression induces a more pronounced lymphatic network, no blood vascular alterations occur. According to Aschen *et al*. (2014), the expression of endogenous VEGFC in transplanted lymph nodes was markedly increased in the perinodal fat and in newly formed lymphatic vessels when compared with control nonoperated lymph nodes. In relation to the Treatment Group, the observed effect on VEGFC gene expression suggest that, as the animals were receiving exogenous VEGFC, they did not produce this protein as effectively as the Surgery Group, suggesting inhibition of endogenous VEGFC production by the animals treated with exogenous VEGFC. However, no literature was found to support this observation, suggesting the need for more investigation. When studying VEGFC/VEGFR3 signalling blockade by neutralization of the VEGFR3, Guo *et al*. (2009) reported increased formation of lymphatic vessels demonstrating a decrease in lymphangiogenesis and lymphatic drainage. These gene expression profiles suggest that the expression of VEGFC and VEGFR3 is more important at the beginning than at the end of the study period for lymphangiogenesis (Carmeliet 2005; Oliver & Alitalo 2005).

As VEGFC and VEGFR3 are biologically fundamental for both angiogenesis and lymphangiogenesis and severe complications occur either by loss of function (e.g., lymphoedema) as a gain of function (e.g., metastases), it is vital to discover tools that adjust VEGFC and VEGFR3 activity and signalling (Bahram & Claesson‐Welsh 2010).

So far, the ability of VEGFC to increase post‐natal lymphangiogenesis has not been universally observed, but both gene and VEGFC protein mediated therapy seem potentially promising (Jin *et al*. 2009). Although further studies in different species are still needed for a better understanding of the neolymphangiogenic action and efficacy of VEGFC, this study offers insights into new methods to promote lymphangiogenesis, focusing on VEGFC as a new target. These findings may contribute to the development of new therapeutic strategies.

## Conclusions

This study showed that there was a significant decrease on the lymphatic vascularization in the left hindlimb of mice after surgical removal of the inguinal lymph node and adjacent lymphatic vessels. Exogenous VEGFC may induce the lymphatic vascularization through the neolymphangiogenesis.

## Source of funding

This work was supported by Fundação de Amparo à Pesquisa do Estado de São Paulo (FAPESP, grant number 2011/04538‐0).

## Conflict of interest

The authors declare that there are no conflicts of interest.

## Ethical statement

The authors confirm that the ethical policies of the journal, as noted on the journal's author guidelines page, have been adhered to and the appropriate ethical review committee approval has been received. The study was approved by the Ethics Committee for the use of Animals of the School of Veterinary Medicine and Animal Science of the University of São Paulo (number 2289).

## Contributions

All authors conceived of the project, performed the experiments, and participated in the analysis. JSPF wrote the first draft of the manuscript,which was proofed and improved upon by PCP.
